# Canadian and English students' beliefs about waterpipe smoking: a qualitative study

**DOI:** 10.1186/1471-2458-9-10

**Published:** 2009-01-10

**Authors:** Jeremy Roskin, Paul Aveyard

**Affiliations:** 1 UK Centre for Tobacco Control Studies, Primary Care Clinical Sciences, University of Birmingham, Birmingham, B15 2TT, UK

## Abstract

**Background:**

Waterpipe smoking is becoming popular among western students. The aim was to understand the appeal to students of this form of smoking when other forms of smoking are becoming less common.

**Methods:**

Waterpipe smokers were identified by snowball sampling and interviewed following a semi-structured schedule in waterpipe cafes and in their homes. Constant comparative analysis was used to derive themes for the analysis.

**Results:**

Waterpipe smokers saw smoking as an alternative to more expensive nights out in bars. The appeal was related to the communal activity and the novelty of the experience. Respondents had not thought deeply about the health risks and reasoned that if no warnings about waterpipe smoking were apparent (unlike cigarette smoking) then it was probably safe. These observations were reinforced by observations about the mildness of the smoke, the fruit flavours, and beliefs about the filtering of the water. Waterpipe smokers felt no pressure to stop smoking and therefore had not tried to do so, but felt it might be something they did not continue after university. Waterpipe smoking was not linked in students' minds to other forms of smoking except in one individual who was using waterpipe smoking to help quit cigarettes.

**Conclusion:**

In the absence of public health information, students have fallen back on superficial experiences to form views that waterpipe smoking is less harmful than other forms of smoking and it is currently much more acceptable in student society than other forms of smoking.

## Background

Waterpipe smoking is a traditional form of tobacco smoking in the Middle East, but appears to have become much more popular in the last twenty years[1]. Anecdotal reports suggest it is now common among western university students and that it may be something that mainly students but not other young people engage in. A recent survey of two American universities found that 15% and 20% had smoked a waterpipe in the past month[2]. In one British university, it was found that 8% of students at one British university were regular waterpipe smokers, with both ever and regular smoking becoming more prevalent with time at the university. Waterpipe smoking was common among students of nearly all ethnic backgrounds[3].

A preliminary survey of waterpipe smokers in the United States who responded to a newspaper advertisement found that the majority were young people and college educated, and most smoked at least monthly and most waterpipe smokers also smoked cigarettes. The large majority felt they were not addicted and could stop easily, though none intended to do so. The large majority thought waterpipe smoking less harmful than cigarette smoking[4]. Results of a survey in one British university were similar, except that only 35% of regular waterpipe smokers also smoked cigarettes[3]. These data however, shed little light on why a somewhat cumbersome method of smoking, which requires more organisation and effort than cigarette smoking, has become popular among western students. A qualitative study was therefore undertaken to understand the beliefs of regular waterpipe smokers.

## Methods

The study took place in Birmingham, England and Toronto, Canada. Patrons of 'shisha cafes', similar in age to students were approached to give an interview, conducted with the aid of a semi-structured interview schedule. Snowball sampling to find other waterpipe smokers (who may not have used cafes) was used. The interview covered initiation of smoking behaviour and frequency, and the social and cultural context in which water pipe smoking occurred. It also concerned knowledge and beliefs with questions including the health consequences of smoking, the content of waterpipe tobacco and smoke, and the opinions of peers and family. The interview schedule aimed to characterise smoking patterns at the start of each session before exploring health beliefs about the practice. For each half of the interview, there were eleven items that the researcher aimed to cover.

The interview was developed and followed advice on structuring interviews and asking questions given by Patton[5]. The lead researcher tested out both the interview schedule and his skills on his friends and colleagues who were waterpipe smokers. These were taped and analysed using Whyte's Directiveness Scale for analysing interview technique[6]. The lead researcher identified points in the interview where a tendency for direct prompting could be minimised to non-verbal cues and reflection on interviewee response. Some minor modifications were then made. Once satisfied with the question schedule and the interview technique, the sampling began. The interviews lasted approximately 30 minutes. All participants provided informed verbal consent. The study was approved by the University of Birmingham Medical School Student Research Ethics Committee.

The interviews were recorded. Detailed notes were made from listening to the recordings including quotations used in the analysis. The notes were checked by listening again to the recordings[7]. Data was analysed using the constant comparison method. Responses were grouped into themes and analysed for connections between the groups. We then crosschecked each theme to ensure data had been adequately categorised. Certain groups of themes were later unified as a single broader theme.

## Results

Twelve interviews were obtained, from thirty people approached; six in England and six in Canada (Table 1). People declined because they did not want to give a lengthy interview or were unwilling to be recorded. The sample size was sufficient to identify and characterise specific themes from those who smoke waterpipes, with no new themes emerging in later interviews. It did not aim to be statistically representative. This is consistent with the principles of qualitative analysis[8]. Nine respondents were students. The interviews took place in early 2007.

**Table 1:** Demographic composition of English & Canadian Interviewees

	English	Canadian	Total
Ethnicity			
White	4	1	5
Arab	1	5	6
Other	1		1

Gender			
Male	4	6	10
Female	2	0	2

Age Group			
18–25	6	5	11
>25	0	1	1

SES			
Student	5	4	9
Employed	1	2	3

Smoking			
Home	4	0	4
Café	0	3	3
Both	2	3	5

Visited Middle East			
Yes	5	6	11
No	1	0	1

### Smoking pattern

Those smoking at home smoked most days, saying that they smoked at home because it was cheaper and more convenient than going out. Café smokers typically smoked once or twice per week, considering these occasions 'an evening out'. In England, smoking in public places was due to be prohibited a few months after the interviews. When asked about the impending ban, most responded that they would continue to smoke at home. In Toronto, indoor smoking in public places was prohibited. However, it was legal to smoke herbal products that contained no tobacco or nicotine. Despite this, three of the six Canadians were smoking tobacco in cafes during the interview. They either supplied their own tobacco or were supplied illegally by the café staff.

### Cultural Context

Cultural and ethnic background was a dominant feature of the context in which waterpipe smoking began. Respondents had either visited or had links to Middle Eastern countries. Most Canadian respondents were Arabic in origin and this was the main influence on waterpipe smoking. In Birmingham, most respondents were not Arabic and waterpipes were seen as part of an alternative cultural view. Waterpipes were seen as exotic and intimate, providing smoker a taste of or reminder of a culture different to their own. These students discovered waterpipe smoking on trips to the Middle East, but knew many others that had not made such trips and yet smoked.

### Social Appeal

Although social context appeared important in initiation, its appeal as a social activity was the dominant explanation for continued use of waterpipes. Respondents from an Arabic heritage saw waterpipe smoking as a means to express that heritage by meeting others from the same background, smoking, playing backgammon, and drinking coffee. For cultural and religious reasons, they would not meet in establishments serving alcohol. Non-Arabic students saw smoking with friends as an affordable and relaxing novelty.

"Friends have been travelling and brought it back; it's something oriental and different to do"^IT3^

Those smoked at home described smoking waterpipes whilst chatting or watching television. Whatever the setting, the waterpipe was a focal point enhancing the social atmosphere. It was an inclusive activity that would be casually commenced and would continue until late at night. For many, it was appealing to be able to smoke intermittently with breaks whilst the pipe was passed round.

"its fun to sit in the lounge, pass round the pipe and each person can smoke as much or as little as they want"^IT3^

Most respondents had friends that disapproved of the waterpipe and did not smoke. Many of those who did not smoke had tried waterpipes previously. Non-smokers would frequently enjoy the social occasion in which the waterpipe was central and enjoy the smell of the smoke. Two respondents smoked alone.

### Relaxation

The enjoyment of the fruit flavours and the experience of inhaling and exhaling large quantities of smooth smoke were the commonest explanations given for its relaxing appeal:

"Breathing smoke in and out from the mouth, I can't really explain it but its somehow subconsciously relaxing."^IT2^

"it is calming, smells nice and I can inhale it as deep as a cigarette if not deeper. I really like to inhale it."^IT3^

### Novelty

It was common to hear descriptions of the production of smoke rings, exhaling in different patterns and slowly inhaling and exhaling to enjoy the flavour. A range of fruit flavours was enjoyed, which seemed to mask the fact that smokers were smoking a tobacco product.

"The strong flavour and strong smoke are great. I can do smoke rings and impress the ladies. There would be no point in smoking if it wasn't flavoured."^IT4^

"The fruit flavour makes it like a candy, it's a silly assumption to make, but it's my assumption."^IT11^

Such assumptions and features have made waterpipe smoking popular amongst young students who may not otherwise use tobacco products.

### Waterpipes and cigarettes

Four respondents smoked cigarettes as well as waterpipe. Many of the waterpipe only smokers felt it was more appealing than cigarettes:

"I don't like cigarettes they stink. The waterpipe leaves no tobacco smell or taste in mouth afterwards. It's like burning incense, its not noxious for those who don't smoke."^IT6^

"I've never smoked cigarettes; the connotations are different to the social fun of the waterpipe."^IT4^

Some were ex-smokers using water pipe as a step down exit from a cigarette smoking habit. They preferred the social atmosphere of waterpipe smoking and felt they wouldn't become addicted to it.

"its cheaper than smoking a pack of cigarettes a day, and there is no easy access like going into a shop or petrol station to buy cigarettes, so I don't spend as much."^IT5^

"I quit cigarettes, shisha is helping me out. It's more of a habit than an addiction."^IT5^

None of the respondents indicated that waterpipe smoking had introduced them to cigarette smoking. One person had tried smoking cannabis through a water pipe but never repeated it.

### Health Beliefs

The texture of the smoke was seen as smoother than cigarettes, which, together with the flavour, made continuous smoking for up to two hours possible. Many associated this more comfortable experience with less harmful health implications:

"It feels light in the throat, not harsh but smooth so I can do a long drag. It means its not hurting my lungs as much or damaging it."^IT5^

"For one hour shisha is healthier. Cigarettes are harder, I cannot smoke 3 cigarettes in an hour." ^IT10^

Some perceived that the smooth texture of the smoke meant they weren't inhaling it despite inhaling for up to ten seconds at a time. To these individuals, the term 'inhaling' seemed to imply the short deep drag delivering smoke into the lungs that they associated with a cigarette. The perception was that waterpipe smoke didn't enter the lungs in the same way.

"You inhale cigarettes but not this. I don't breathe deep; I breathe it into my mouth and then out. I don't inhale."^IT4^

When asked how healthy waterpipe smoking was compared to cigarettes, nine respondents considered it to be less harmful, two considered the two methods of smoking to be equivalent, and one person considered waterpipe smoking more harmful. Those who considered waterpipes less harmful included all respondents that have never smoked cigarettes. This may account for their willingness to participate in one method of smoking but not the other.

"Fruit flavour makes it less harmful. I don't believe it's as harmful as cigarettes. Everyone seems to believe this, that it's less toxic. I know students who smoke shisha but wouldn't smoke cigarettes. If offered a cigarette, they'd turn it down."^IT1^

When the basis of this belief was challenged, most attributed its origin to common perception amongst their friends: "it's what people say,"^IT3 ^"from what I've been told."^IT1^

English respondents described the media campaigns warning about the dangers of cigarette smoking, which they believed. The lack of similar campaigns about waterpipes implied they must be safer.

"There are no warnings on TV, if there were warnings, I'd be more aware."^IT3^

"Cigarettes are much more harmful as the dangers are publicised, I don't really see the danger [of waterpipes]."^IT4^

Most lacked any knowledge about the health implications of waterpipes. The single respondent who believed waterpipe smoking to be more harmful had a background in scientific education and his beliefs were based upon the opinion of a toxicology teacher:

"In shisha...the way it's prepared with sugar in tobacco and charcoal, glycerine burns in the head producing free radicals. These are very harmful and causing cancers."^IT7^

### Knowledge about waterpipe tobacco

When asked about the content of the tobacco and the function of the different components of the waterpipe, most understood how the pipe produced the smoke. Four respondents, all from Canada, had a degree of knowledge about the composition of the tobacco. They named the chemicals in tobacco and these people saw waterpipes as equally or more harmful than cigarettes. No English respondents had the same knowledge or beliefs.

Most saw the charcoal that burns the tobacco as potentially harmful because it was a synthetic product. Any harm or ill health caused by waterpipe smoking was attributed to the charcoal rather than the tobacco.

"I guess it [charcoal] must be quite damaging to health because it burns; maybe it's where the tar comes from."^IT3^

"It's a chemical based product containing benzene or whatever."^IT6^

A few saw the charcoal, which sparkles when initially ignited, with a positive view:

"It's a novelty aspect isn't it."^IT2^

"Its like using a BBQ when cooking food to eat, as long as you don't overcook."^IT11^

A few alluded to the water in the base of the water pipe as having filtering properties. The same individuals considered water pipe smoking to be a relatively healthier behaviour:

"From what I've been told, the water acts as a filter to get rid of bad stuff."^IT4^

"Water catches the ashes from the charcoal. Not like a cigarette filter, water is a natural filter."^IT5^

Respondents were shown a packet of Naklah tobacco, a product produced in Egypt and used in waterpipes globally. Written on the side of the packet is 0% tar, 0.5% nicotine.

All of the English respondents were unfamiliar with anything written on the tobacco box:

"The packets tend to be in Arabic, so I don't read them."^IT1^

The others saw it as a positive health statement, reinforcing their beliefs that smoking water pipe was safer than cigarettes:

"Tar is what is bad for you so this must be healthier."^IT3^

Only one respondent had ever seen these figures before. This respondent considered it as a health warning. Others were unsure how to interpret these 'facts':

None of the respondents were concerned about the nicotine levels in this product. Whilst some respondents felt they craved the relaxing experience of smoking a waterpipe no one identified this as having an addiction.

Canadian herbal molasses smokers considered their smoking to be harmless. When shown the waterpipe tobacco, they concluded that their herbal preparations were completely healthy:

"The harm is the nicotine. Herbal is 100% healthy. It's just a smoke, its not addictive, it's clean."^IT9^

"There are not as many additives [in Naklah tobacco] as cigarettes. It seems cleaner. If real tobacco has no tar, herbal must be even safer."^IT10^

### Health Problems

Half mentioned past health problems after prolonged smoking. Interestingly, all those who mentioned such occurrences were respondents that saw smoking as harmless. The mildest problem reported was a head rush, noticed more when smoking for a long time, or when smoking for the first time in a while. Some smokers identified this as a positive experience. Others mentioned various throat problems after sessions lasting more than an hour. These were commonly a harsh sensation in the throat, associated with a decline in the quality of the smoke towards the end of the session.

"After a long time, it stops being enjoyable – my throat feels tight, like its closing up a little bit. It depends on the coal, if it's been on too long."^IT2^

"After two hours I feel the pain. I know my limits."^IT6^

### Family Acceptance

In Canada, those that smoked in cafes reported their families disapproved of smoking at home. Smoking at home was rare and done outside. Some English respondents smoked in front of their families without meeting disapproval. In some instances parents had joined in. Respondents did not believe they would have the same experience with cigarette smoking:

"I have never smoked cigarettes in front of my parents. They'd be surprised, annoyed and disappointed. There is something more acceptable about shisha. Maybe it's a myth."^IT1^

"When I explained to my parents it was harmless, they were fine. They've tried it."^IT2^

### Desire to quit or change

Most of the students felt they would continue the same pattern whilst at university but might reconsider in the future. Some respondents said it would depend on the relative harm:

"If it was like 1 cigarette I'd carry on. If it was like a pack a day I might think about it."^IT2^

"If I had good facts to show it was bad, I'd probably cut down but not completely stop."^IT4^

Others were less willing to consider a change in habit, should information suggest that waterpipe smoking was unhealthy. This seemed to stem from a strong belief based on their experiences that waterpipe smoking was relatively benign.

"If I felt unhealthy I would stop. When I smoked cigarettes, I sometimes felt out of breath and knew the cigarettes were harming me. I don't feel that shisha is harming my body."^IT6^

"If everything in life is harmful, we might as well not be here. I need things to enjoy in life. I enjoy this so I'd continue." ^IT7^

## Discussion

Waterpipe smoking is a common part of student experience in England and in the USA[2,3]. These results suggest that Middle Eastern cultural heritage has played a role in introducing the waterpipe to student culture, but that it is passing from student to student for other reasons. Part of the popularity is because waterpipe smoking is seen as considerably less harmful than cigarette smoking. In part, these beliefs stem from the flavouring of the tobacco, but mainly from a commonly held belief of uncertain origin. It is not due to the misleading description on the tobacco packages. Waterpipe smoking offers students a social means to get a mild 'rush', which is affordable compared to bars and restaurants, and it does not exclude non-smokers, who enjoy the experience because the smoke is much more acceptable than cigarette smoke.

Epidemiological studies show evidence that waterpipe smoking is associated with an increased risk of cardiovascular disease and cancer and with proxy markers of the risk of developing these[9,10]. However, compared to the wealth of information about the health hazards of cigarette smoking, there is insufficient information about the corresponding hazards of waterpipe smoking[11,12]. We did not enquire specifically about whether the students thought they would continue smoking waterpipe use for many years, but the hazards of long-term health consequences are mainly relevant to people that assume they will use it long-term. The students here had not thought deeply about these risks and relied on trivial observations, like the fruit flavour, to support the widely held belief that waterpipe smoking was harmless. A Cochrane review of interventions to encourage waterpipe smokers to stop found no studies on whether health education was effective or not[13]. However, all respondents reported some of their friends disapproved of waterpipes. This disapproval did not seem to dent smokers' beliefs that waterpipes were benign and was not so strong that the non-smokers absented themselves. The beliefs in the benign nature of waterpipes were also not disturbed by apparent minor health effects. In some cases, these effects were seen as less than the comparable effects of cigarette smoking, but this was true of a minority.

We do not really know whether tobacco addiction is more common among regular cigarette smokers than among regular waterpipe smokers because this has not been fully investigated, but some evidence suggests that cigarette smoking might be more addictive than waterpipe smoking[11,14]. Perhaps the most important manifestation of addiction is difficulty stopping using waterpipes should a person choose to do so[15]. The problem with waterpipe smoking in students is that so few have tried to stop that we cannot tell how commonly this happens. None of these respondents felt trapped by smoking.

Waterpipe smoking is most visible to the public in shisha cafes. In Birmingham, where this study took place, several but not all of these have closed since the ban on indoor public smoking came into force. In Canada, despite the legislation, tobacco smoking in waterpipes continued. However, home smoking was clearly established, was the most common form of smoking in Birmingham[3], and it could be that closing the cafes will not greatly reduce the prevalence of waterpipe smoking. Over half of American students had their first experience of waterpipe smoking at someone's home, not in a café[16]. Waterpipe smoking provided these students with an opportunity for a cheap social evening, which would not be influenced by restrictions on smoking in public. This perhaps emphasises the need to tackle waterpipe smoking by health education rather than rely on clean air legislation alone.

There were some limitations to this study. The aim was to study a specific population group, student waterpipe smokers. Such smokers are not easy to find and hence a method of snowball sampling was used rather than some theoretically based purposive sampling. This could have led to sampling a particular subculture of waterpipe smokers. Purposive sampling might have uncovered other smokers with different beliefs and experiences. The study did not sample sufficient smokers that used both cigarettes and waterpipes and there was insufficient time to delve into the inter-connections between both forms of smoking. One person reported using waterpipe smoking to help him stop smoking. The interviewer did not enquire whether waterpipe smoking had led onto cigarette smoking in those smoking both products. Understanding the role waterpipe smoking has in the 'tobacco economy' is important in assessing the public health significance of this behaviour. In this study and in a similar survey of smokers, most waterpipe smokers felt that this form of smoking was less dangerous than cigarette smoking[3]. Only one person in this study expressed the belief that waterpipe smoking was more dangerous than cigarette smoking. There was not sufficient time to enquire about why he continued to smoke waterpipes and had not switched to cigarettes and what waterpipes might offer instead of cigarettes. It is also worth noting that most people approached to participate declined to do so, which might mean the sample was biased. However, there is no good reason to assume that beliefs about waterpipe smoking and the role it played in a person's life affected decision to participate, which was mainly down to availability of time to volunteer for the study.

These data have implications for public health. Part of the reason for the beliefs about the comparative healthiness of waterpipe smoking compared to cigarette smoking is that there are no warnings discouraging the former unlike the latter. Students relied on superficial differences in the taste and smell of smoke and the water filtration to base their beliefs upon, but they mostly appeared to come from what was perceived as generally true. These beliefs might be changed by simple presentation of toxicological and epidemiological evidence to the student population, as summarised in recent publications. The effects this might have on waterpipe smoking is unknown. There is a debate in public health circles about whether the use of some forms of tobacco use might be helpful and not harmful; for example the use of snus. Waterpipe smoking involves burnt tobacco, and is therefore likely to be considerably more harmful than snus, but whether overall it is as addictive or as harmful as cigarette smoking is unclear. Longitudinal studies of students would be needed to examine the inter-relationships between different forms of tobacco consumption.

## Conclusion

Waterpipe smoking in students is established by connections with the Middle East but has become seen as an affordable and enjoyable social activity with few health risks. It is used by a few as a means to cease smoking cigarettes. How important the epidemic of waterpipe smoking among students will be to public health is still uncertain, but it is likely that it would be curbed by authoritative information about potential harm. The current lack of health information is currently viewed as tacit official acceptance of waterpipe smoking.

## Competing interests

Paul Aveyard had served as a consultant to and received funding from McNeill AB, Xenova Biotechnology, and Pfizer for work on smoking cessation.

## Authors' contributions

JR initiated the study and it was planned by both authors. JR carried out the study and both he and PA analysed the data. JR wrote the first draft and PA amended it. Both authors read and approved the final manuscript.

## Pre-publication history

The pre-publication history for this paper can be accessed here:


